# Merkel Cell Carcinoma of the Eyelid and Periocular Region

**DOI:** 10.3390/cancers6021128

**Published:** 2014-05-09

**Authors:** Helen Merritt, Matthew C. Sniegowski, Bita Esmaeli

**Affiliations:** 1 Orbital Oncology and Ophthalmic Plastic Surgery Program, Department of Plastic Surgery, The University of Texas MD Anderson Cancer Center, Houston, 1515 Holcombe Blvd, Unit 1488, Houston, TX 77030, USA; 2 Ruiz Department of Ophthalmology and Visual Science, The University of Texas Medical School at Houston, Houston, TX 77030, USA

**Keywords:** Merkel cell carcinoma, eyelid, periocular

## Abstract

Merkel cell carcinoma (MCC) in the eyelid and periocular region can be treated surgically, in most cases, with preservation of the eye and reasonable visual function. Adjuvant radiation therapy, sentinel lymph node biopsy, and chemotherapy should be considered for MCC of the eyelid and periocular region, especially for larger tumors that are T2b or more advanced and lesions that present with regional nodal or distant metastasis.

## 1. Introduction

Merkel Cell carcinoma (MCC) is a rare and aggressive neuroendocrine malignancy of the skin with approximately 1,500 new cases diagnosed each year [1]. Diagnosis most commonly occurs after the sixth decade with age-specific rates highest over the age of 70 years [2,3,4]. The large prospective and retrospective studies have shown a slightly higher prevalence in men than women [2,3,4,5,6]. Over 95% of recorded cases of MCC as of 2010 occurred in Caucasians, with a much lower incidence in other ethnicities [3,5].

### 1.1. History

The cell of origin was first described by Friedrich Merkel in 1875 as epithelial in derivation with neuroendocrine differentiation and has been hypothesized to function as a tactile mechanoreceptor. Toker in 1972 first described MCC, referring to it as “trabecular carcinoma of the skin” [7]. It has since been suggested that these tumors may instead derive from proliferations of dermal pluripotent stem cells without mechanoreceptor function having similar electron micrographic appearance and similar staining for characteristic neurofilaments and cytokeratins as Merkel cells [8].

### 1.2. Clinical Presentation and Associations

**Figure 1:**
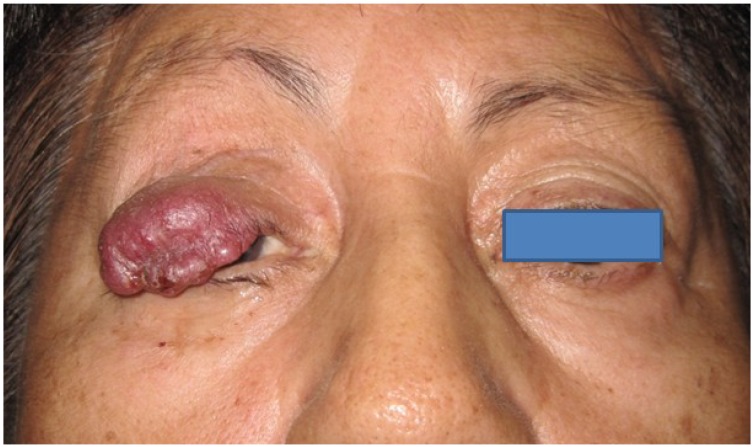
External photograph in a patient with typical appearance of Merkel cell carcinoma of upper eyelid.

Merkel cell carcinoma lesions typically appear as asymptomatic, solitary nodules with distinctive pink, red or violaceous coloring and may have overlying ulceration or telangiectasia (Figure 1) [4,9]. There is a high association of MCC with history of sun exposure, infection with polyomavirus, and immunosuppression [4,10,11] In particular, the relative risk of MCC development in those with concurrent HIV infection has been found to be 13.4 [12]. Additionally, MCC has been associated with concurrent malignancies and may share carcinogenic processes with other cells of neural crest origin [13].

### 1.3. Histopathology

Microscopic evaluation of periocular and eyelid MCC reveals findings consistent with MCC in other anatomic sites including poorly-defined groupings of small cells in the dermis and subcutaneous tissue with characteristic scant cytoplasm and salt-and-pepper, finely dispersed chromatin [14]. Electron microscopy can aid in diagnosis, demonstrating characteristic cytoplasmic granules 80 to 150 nm in diameter [9,15]. The immunohistochemical presence of neurofilament protein, cytokeratin 20, and neuron-specific enolase along with the absence of leukocyte common antigen and S100 protein are helpful for histopathological differentiation from similarly appearing epithelial and neuroendocrine tumors [9,16,17].

## 2. Merkel Cell Carcinoma of the Eyelid

46%–48% of all Merkel cell carcinomas appear in the head and neck region [3,5]. Of the tumors presenting in the head and neck, the eyelids are common primary sites with incidence between 5% and 20% of all cases of head and neck Merkel cell carcinoma [3,5,9,18].

MCC is more commonly identified in the upper eyelid and usually arises near the eyelid margin, often causing partial or complete eyelash loss [9,16,19,20]. The typical appearance is that of a violacious, rapidly growing mass lesion associated with soft tissue ulceration, destruction, and eyelash loss (Figure 1). The clinical behavior and epidemiologic features of eyelid MCC are similar to MCC in other anatomic sites; however, a review of 89 cases reported in the literature prior to 2006 suggested a higher incidence in women than men [16]. Merkel cell carcinoma in the eyelid area is commonly misdiagnosed initially as cysts, chalazia, or basal cell carcinomas [4,19].

## 3. Staging of Merkel Cell Carcinoma

### 3.1. America Joint Committees on Cancer (AJCC) Staging

Tumor size at presentation is an important prognostic factor for periocular and eyelid Merkel cell carcinoma. The 2010 7th edition of the AJCC Cancer Staging Manual uses tumor size to stage MCC, categorizing based on the smallest T category consisting of lesions measuring 2 cm or less, but lacks site-specific prognostic guidelines for MCC of the eyelids [21]. Analysis of 4,376 patients with MCC of the head and neck found that 69% of tumors presenting in this region were smaller than 2 cm at the time of diagnosis [5].

A recent retrospective investigational analysis of 18 patients treated for MCC of the eyelid and periocular region at MD Anderson Cancer Center concluded that while the AJCC T for Merkel cell carcinoma significantly correlated with category disease-free survival in patients with periocular MCC, the eyelid carcinoma T category showed a better correlation with disease free survival when staging MCC of the eyelids as it discriminates cases within the MCC T1 category thereby giving more specific prognosis for lesions smaller than 2 cm [22]. Table 1 summarizes the classification scheme for AJCC 7th edition both for eyelid carcinoma and for Merkel cell carcinoma. The N and M categories which categorize nodal and distant metastasis respectively, are the same for the eyelid carcinoma and Merkel cell carcinoma staging in AJCC.

### 3.2. Sentinel Lymph Node Biopsy

Sentinel lymph node (SLN) biopsy has been shown to be an appropriate method for detecting regional lymph node disease and should be considered for periocular MCC [23,24]. The incidence of reported sentinel lymph node (SLN) positivity in large trials of patients with MCC carcinoma of all sites ranges from 23% to 45% [23,25,26]. A study of 95 patients with MCC undergoing SLN biopsy revealed greater likelihood of positive results with increased tumor size, higher tumor thickness, high mitotic rate, and infiltrative growth pattern [23].

**Table 1:** Comparison of American Joint Committee on Cancer, 7th edition, T Categories for Eyelid Carcinoma and Merkel Cell Carcinoma.

	Merkel Cell Carcinoma		Eyelid Carcinoma
TX	Primary tumor cannot be assessed	TX	Primary tumor cannot be assessed
T0	No evidence of primary tumor (e.g., nodal/metastatic presentation without associated primary)	T0	No evidence of primary tumor
Tis	In situ primary tumor	Tis	Carcinoma *in situ*
T1	Less than or equal to 2 cm maximum tumor dimension	T1	Tumor 5 mm or less in greatest dimension, not invading the tarsal plate or eyelid margin.
T2	Greater than 2 cm but not more than 5 cm maximum tumor dimension	T2a	Tumor more than 5 mm, but not more than 10 mm in greatest dimension, or, any tumor that invades the tarsal plate or eyelid margin
T3	Over 5 cm maximum tumor dimension	T2b	Tumor more than 10 mm, but not more than 20 mm in greatest dimension, or, involves full thickness eyelid
T4	Primary tumor invades bone, muscle, fascia, or cartilage	T3a	Tumor more than 20 mm in greatest dimension, or, any tumor that invades adjacent ocular or orbital structures. Any T with perineural tumor invasion.
		T3b	Complete tumor resection requires enucleation, exenteration, or bone resection
		T4	Tumor is not resectable due to extensive invasion of ocular, orbital, craniofacial structures, or brain

This study found that 68% of patients with MCC larger than 2 cm had positive SLNs. Furthermore, the same study revealed that 23% of patients with MCCs less than 1 cm have positive SLNs, which is still a high enough rate of positivity to justify SLN biopsy even for lesions less than 1 cm in size [23]. Review of 2194 patients with head and neck MCC showed a 70% increased risk of mortality in patients who did not have nodes histopathologically examined at the time of diagnosis in comparison to those biopsied and found to have nodes free of disease [5]. As a proof of principle, our group has previously reported patients with Merkel cell carcinoma of eyelid who had positive SLNs [27]. In our recent study of 18 patients with Merkel cell carcinoma the overall proportion of patients who presented with lymph node metastasis—whether based on clinical examination, on radiographic images, or based on SLN biopsy—was 22% [22]. Three of four patients with nodal metastasis at presentation had >T2b tumor, 2 additional patients developed nodal metastasis during the follow-up period. These trends suggest a need to address the regional lymph nodes in patients with Merkel cell carcinoma of eyelid and periocular region. Based on this study it was felt that multidisciplinary management of eyelid MCC is essential with strong consideration for either prophylactic radiation therapy or sentinel lymph node biopsy, and selective lymph node dissection in patients with either node positive disease or tumors that are T2b or more advanced at presentation by AJCC T category for eyelid carcinoma.

## 4. Prognosis

Merkel cell carcinoma of all sites has a poor prognosis due to commonly delayed diagnosis, rapid and aggressive growth, early nodal and distant metastases, and high incidence of local recurrence [2,5]. MCC of the head and neck has been found to be particularly aggressive with higher likelihood of bone, cartilage, and skeletal muscle invasion when compared to MCC of other sites [5]. While eyelid and periocular lesions may present earlier due to better visualization of the lesion in this location, MCC of the head and neck still demonstrates high incidence of spread to the regional lymph nodes with up to 2/3 of patients having regional lymph node metastasis and 1/3 of patients with distant metastasis within 18 months of diagnosis [9,16,19]. MCC of the eyelid has been reported to have an overall metastatic rate ranging from 10% to 30% [16,19,20], regional lymph node recurrence rate of 20%, and distant metastasis rate of 5% [20]. The recent study by our group demonstrated a 22% metastatic rate overall which included an 11% nodal metastasis and 11% distant metastasis rate [22]. Within this study the only statistically significant factor associated with a decreased disease free survival was nodal involvement (metastasis) at presentation.

Distant metastasis from Merkel cell carcinoma of all anatomic sites most commonly occurs in the skin, bone, brain, liver, and lung [3,5,9]. The reported rate of distant metastasis is as high as 36% [28] to 38% [19]. Survival analysis of 2104 patients with head and neck MCC showed an association between an increased risk of death and the characteristics of male sex, tumor size between 2 and 5 cm, tumor extension beyond the dermis, and presence of nodal or distant metastasis [5].

## 5. Treatment

### 5.1. Surgical Excision

The treatment of MCC is largely empiric as there are no prospective, randomized studies investigating management in the literature to date [6,22]. The standard-of-care treatment of MCC of all sites includes initial wide surgical excision with margins from 1–3 cm [6,29], however for eyelid and periocular Merkel cell carcinomas such wide margins are not practical and would yield sacrifice of the eye each time. For eyelid Merkel cell carcinomas, a more conservative approach with surgical margins as small as 5 mm is acceptable [19]. Mohs micrographic surgery has been shown to be an appropriate approach to ensuring margins free of disease; however, wide excision with frozen section control of margins may also be used for eyelid MCC’s since the goal is not just to achieve negative margins but also have at least a 5 mm safety margin [9,19]. Due to the aggressive biology of Merkel cell carcinoma and its exquisite radiosensitivity, postoperative adjuvant radiation therapy is often considered and done for cases of eyelid MCC as long as there are no contraindications to radiation therapy. In our recent series of 18 periocular MCC which may be the largest single-center case series of periocular MCC published to date, 17 (94%) of 18 patients had postoperative adjuvant radiation therapy after conservative negative surgical margins were achieved [22]. Due to frequent regional lymph node involvement, prophylactic lymph node dissection has been suggested but it is a fairly extensive surgery that may not improve loco-regional control nor improve overall disease survival [5]. Sentinel lymph node biopsy, however, is a more selective biopsy of only a few lymph nodes that are mapped to drain the lesion and has been shown to be a sensitive method to detect microscopic regional lymph node involvement. Complete nodal dissection and/or radiation therapy can be performed for nodal basins that harbor a positive SLN [5,22].

### 5.2. Radiotherapy

The role of adjuvant or prophylactic radiotherapy in the treatment of MCC remains somewhat controversial. While the literature clearly demonstrates that adjuvant radiotherapy to the surgical bed improves loco-regional control, there is not strong evidence that adjuvant radiotherapy to the surgical bed alone improves survival [28,30,31]. Regarding radiotherapy to the regional lymph nodes the role of adjuvant radiotherapy remains controversial. Owing to the reported 24% to 68% risk of nodal metastasis for MCC, some practitioners may justify prophylactic irradiation of the regional lymph nodes in all patients [6,23]. While other practitioners may advocate nodal bed irradiation only if SLN biopsy proves positive due to the fact that all draining lymph node basins (parotid, submandibular, cervical) must be included in the prophylactic radiation field [22,26,30]. Yet despite these controversies, there are several studies in the literature demonstrating improved overall survival with adjuvant radiotherapy to the surgical bed and draining lymph node basins [31,32,33,34,35].

### 5.3. Chemotherapy

Systemic chemotherapy has been implemented in the treatment of MCC and may be considered for patients with distant metastasis or those with extensive regional nodal metastasis [36,37,38]. MCC has demonstrated sensitivity to drugs such as cisplatin, cyclophosphamide, doxorubicin, vincristine, and 5-fluorouracil [30,36]. Adjuvant chemotherapy, however, has not been proven to diminish the rates of recurrence nor improve survival [37]. A retrospective review of 107 patients with locally advanced or metastatic MCC showed overall clinical response rate to first-line chemotherapy of 61%, however this study additionally demonstrated high toxicity-related mortality [36]. A recently published series of 4 patients with metastatic MCC has shown promising results with the use of oral etoposide which demonstrated a 100% disease control rate at an average follow up on 14 months, while being well tolerated [38].

### 5.4. Alternative Therapy

Mechanism-based therapies are also being currently explored as further adjuvant treatment options for MCC including somatostatin analogues (octreotide), receptor tyrosine kinase inhibitors (pazopanib), PI3KCA inhibitors, and drugs targeting apoptotic protein surviving and CD56 [39]. Conversely, investigations into the role of the MAP kinase pathway and B-Raf mutations in the etiology of MCC, two factors largely implicated in the pathogenesis of melanoma and cutaneous carcinoma, suggest no similar correlation with MCC tumorigenesis [1]. A promising relationship had been demonstrated between MCC pathogenesis and the bel-2 proto-oncogene (bcl-2), a regulator of cell apoptosis [40], which promised treatment option expansion [1]. A 2009 phase II trial of bcl-2 antisense therapy in advanced MCC, however, showed no objective responses despite excellent toleration of the drug [41].

### 5.5. Spontaneous Complete Regression

Within the literature there are also rare reports of spontaneous complete regression of metastatic MCC. While there are fewer than 20 reported instances of complete regression of MCC in the literature, several studies have evaluated the histopathology of these spontaneous resolution sites and have demonstrated extensive fibrosis and chronic inflammatory cell infiltration, including T-cells. These findings have led to the conclusion that, similar to cutaneous melanomas, T-cell immunity, likely plays arole in spontaneous regression of some cases of MCC [42].

In conclusion, Merkel cell carcinoma is a rare but aggressive tumor that can be encountered on the eyelid and in the peri-ocular region. Uniform staging with AJCC 7th edition TNM staging for eyelid carcinoma along with prospective multi-institutional study will likely clarify best treatment practices in the future.
